# Geographic Distribution and Mortality Risk Factors during the Cholera Outbreak in a Rural Region of Haiti, 2010-2011

**DOI:** 10.1371/journal.pntd.0003605

**Published:** 2015-03-26

**Authors:** Anne-Laure Page, Iza Ciglenecki, Ernest Robert Jasmin, Laurence Desvignes, Francesco Grandesso, Jonathan Polonsky, Sarala Nicholas, Kathryn P. Alberti, Klaudia Porten, Francisco J. Luquero

**Affiliations:** 1 Epicentre, Paris, France; 2 Médecins Sans Frontières, Geneva, Switzerland; 3 Ministère de la Santé Publique et de la Population, Cap Haitien, Haiti; Massachusetts General Hospital, UNITED STATES

## Abstract

**Background:**

In 2010 and 2011, Haiti was heavily affected by a large cholera outbreak that spread throughout the country. Although national health structure-based cholera surveillance was rapidly initiated, a substantial number of community cases might have been missed, particularly in remote areas. We conducted a community-based survey in a large rural, mountainous area across four districts of the Nord department including areas with good versus poor accessibility by road, and rapid versus delayed response to the outbreak to document the true cholera burden and assess geographic distribution and risk factors for cholera mortality.

**Methodology/Principal Findings:**

A two-stage, household-based cluster survey was conducted in 138 clusters of 23 households in four districts of the Nord Department from April 22^nd^ to May 13^th^ 2011. A total of 3,187 households and 16,900 individuals were included in the survey, of whom 2,034 (12.0%) reported at least one episode of watery diarrhea since the beginning of the outbreak. The two more remote districts, Borgne and Pilate were most affected with attack rates up to 16.2%, and case fatality rates up to 15.2% as compared to the two more accessible districts. Care seeking was also less frequent in the more remote areas with as low as 61.6% of reported patients seeking care. Living in remote areas was found as a risk factor for mortality together with older age, greater severity of illness and not seeking care.

**Conclusions/Significance:**

These results highlight important geographical disparities and demonstrate that the epidemic caused the highest burden both in terms of cases and deaths in the most remote areas, where up to 5% of the population may have died during the first months of the epidemic. Adapted strategies are needed to rapidly provide treatment as well as prevention measures in remote communities.

## Author Summary

In October 2010, a large cholera outbreak was declared in Haiti and rapidly spread throughout the country, quickly overwhelming the existing health system. Specialized treatment structures were opened rapidly, generally in cities or large villages, and decentralized treatment units or rehydration points were gradually opened later on. To gain insight into the true burden of the cholera outbreak in the community and on potential geographical differences due to accessibility, we conducted a survey in April–May 2011 in a large rural area across four mountainous districts in the Nord department. We interviewed 3,187 households, corresponding to 16,900 individuals, of whom 2,034 (12%) had had diarrhea, probably cholera, since the beginning of the outbreak. The two most remote districts showed higher proportions of population affected by the disease, up to 16.2%, and higher proportions of deaths among patients with probable cholera, up to 15.2%, than the two districts with better accessibility. Remote populations, older patients, severe cases and those not seeking care were at increased risk of dying of the disease. These results show the very high burden of the cholera outbreak in remote areas, emphasizing the need to develop strategies to rapidly provide treatment and prevention measures in remote communities.

## Introduction

The cholera epidemic in Haiti, which began in 2010 spread rapidly in both urban and rural areas. One month after confirmation of the first case in Mirebalais, in the department of Centre, the whole country had been affected [1,2]. During the first few days, the focus was on case management in hospitals, which were quickly overwhelmed [1]. Gradually, the Ministry of Health (MSPP), together with partners including non-governmental organizations (NGOs), started setting up dedicated treatment facilities ranging from large specialized centers to decentralized oral rehydration points (ORP) in more isolated communities, cholera-specific health education messages, and water and sanitation activities [1,3]. A national training program for cholera management was developed to train clinical staff, nearly all of whom were unfamiliar with the disease [4]. Despite these efforts, over 600,000 cases of cholera and 7,000 deaths were reported by the national health-structure based surveillance system within two years of the first case [5], and, at the time of writing this article, cases are still being reported (http://mspp.gouv.ht/).

**Fig 1:**
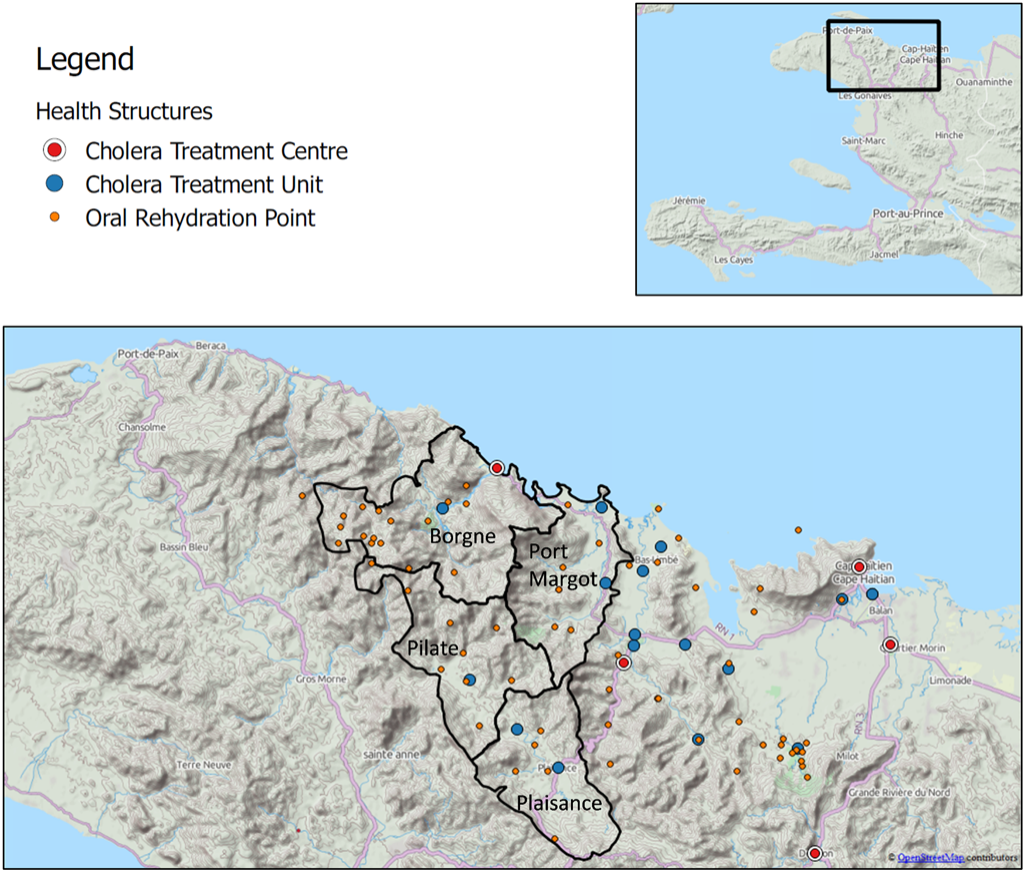
The districts of Plaisance, Pilate, Borgne, Port Margot, and distribution of cholera treatment structures, Nord Department, Haiti, November 2010-March 2011. CTC: cholera treatment center; CTU: cholera treatment unit; ORP: oral rehydration point.

In the Nord department, the first cholera cases were officially reported on October 22^nd^, 2010 (week 42). The first cholera treatment center (CTC) was opened on October 23^rd^ in Cap Haitien, the administrative center of the department, and cholera treatment units (CTUs) were gradually opened in November in the main communes of the department. ORPs only started operating in remote areas in December 2010 and January 2011 (Fig. 1).

Since national surveillance data were based on reports from the health structures and were likely to miss community cases, large retrospective population-based surveys were conducted by Médecins Sans Frontières (MSF) in April and May 2011 to estimate the cholera burden during the first weeks of the epidemic and get insight into health-seeking behavior. Here, we present the results of a survey that was conducted in a large rural, mountainous area across four districts of the Nord department, chosen to facilitate comparison between regions with good versus poor accessibility by road, and with rapid versus delayed response to the outbreak. We also present results of a risk factor analysis carried out in the same area, looking for potential explanatory factors for geographic differences in mortality with the aim of providing information to improve future outbreak response strategies in similar settings.

## Methods

### Ethics statement

The procedures followed were in accordance with the ethical standards of the Helsinki Declaration. The National Ethics Committee of Republic of Haiti granted ethical approval and the Ministry of Public Health of the Republic of Haiti gave authorization to perform the survey. Written informed consent for study participation was obtained from all participants.

### Study setting

The Nord department is located on the northern coast of Haiti and encompasses coastal and mountainous areas, with limited road infrastructure (Fig. 1). Health structures are located in urban centers with catchment areas of hundreds of square kilometers. Many houses are not accessible by road and some hamlets are located more than a 10-hour walk from the nearest health structure. The study took place in 2011 in four districts (“communes”) of the Nord department: Plaisance, Pilate, Borgne and Port Margot (Fig. 1).

Villages in the districts of Plaisance and Port Margot are mainly accessible by roads, while villages in the more mountainous districts of Pilate and Borgne are more difficult to reach due to their mountainous terrain. Each district is divided into 6 to 8 sections. According to a 2009 estimate [6], the total population in the four districts was 218,649 inhabitants, of whom 173,903 lived in rural areas targeted by this survey.

From the beginning of the cholera outbreak and until the time of the survey, 29,295 cases and 654 deaths were reported in the Nord department [Rapport journalier MSPP du 22 mai 2011], for an estimated attack rate (AR) of 2.9% and case-fatality rate (CFR) of 2.2%.

MSF, one of the main partners working with the MSPP to treat cholera patients in Haiti, intervened early in Plaisance, where a CTU was opened in epidemiological week 44, 2010, followed by a CTU in week 47 and 5 ORPs in weeks 49 and 50. In Pilate, the intervention was slightly delayed, with a CTU opening in week 47, followed by 2 ORPs in week 49 and another 6 ORPs in week 1, 2011. In Borgne, a CTC opened in week 47, followed by a CTU in week 50, 5 ORPs in week 52, 9 ORPs in week 1, 2011 and 6 ORPs in week 2. In Port Margot, a CTU was opened by the Catholic Church before week 48 and an ORP by the MSPP in week 48, while MSF started late, with one ORP in week 52, 2 ORPs in week 1, 2011 and a CTU and one additional ORP in week 2.

### Study design

A two-stage, household-based cluster survey was conducted in the study area. The sample size was 16,000 individuals, calculated to estimate an expected crude mortality rate of 0.5 per 10 000 persons per day with a precision of 0.1, an anticipated design effect of 2 and a recall period of 170 days (from October 17^th^, 2010 to the earliest survey date). In total, 140 clusters of 23 households, with an expected average size of five members per household, were selected in the four districts.

For the first sampling stage, clusters were attributed to each communal section proportionally to the size of the rural population (37 in Plaisance, 34 in Pilate, 42 in Borgne and 27 in Port-Margot). Villages of more than 5,000 inhabitants were considered urban and therefore excluded from the sampling frame. For the second stage, a large number of random geographic points was generated using the R statistical package. These randomly selected points were then mapped using Google Earth; only points with a house found visually within a 50m radius were retained. For each section, the number of points allocated was then randomly selected from the remaining points. The corresponding GPS points were used in the field to locate the initial house of each cluster. The next house was selected by proximity, i.e., next closest house, until 23 households had been visited in each cluster. Households in which no adult was present at the time of the first visit were revisited at least once before the study team left the village.

### Data collection

Data were collected using a standardized questionnaire. After providing written consent, the head of household was asked to provide the age and sex of all household members (defined as persons living under the same roof and sharing meals). For each household member present at the beginning of the recall period, the head of household was asked about episodes of diarrheal illness (defined as at least three watery stools within a 24-hour period) and deaths that occurred during the recall period. More detailed information was collected on the diarrheal episode, or on the most severe one if multiple episodes were reported for the same individual. Information collected included duration and symptoms of the episode, health-seeking behavior (i.e., type(s) of health structure(s) visited or reason for not visiting a health structure), and outcome (i.e., death or survival). Severe cases were defined as those in which patients reported lethargy or altered consciousness during the diarrheal illness. Death was considered related to diarrhea when it was reported as the outcome of the most severe diarrheal episode.

In each cluster, the time and type of transport to the closest village with a health structure (excluding ORPs) was documented.

### Statistical analysis

Double data entry was done in Epidata 3.1 (EpiData, Odense, Denmark) by four trained data entry clerks. Data validation and statistical analysis were performed using Stata 11 (StataCorp, College Station, Texas, USA) and R Statistical Software. As not all clusters achieved a sample of 23 households, weighted analysis was used to adjust for the probability of each household being selected, by dividing the expected household number per cluster (23) by the actual number of households included. In all analyses, we accounted for the clustering of households within the cluster and applied the selection weights. Design effects are reported where relevant.

Each principal outcome was presented as a percentage with its associated 95% confidence intervals. Results were then extrapolated to the overall rural population by applying a weight which multiplies the selection weight by the total rural population of each district divided by the sample size in this district. A geographical representation of each principal indicator was done using a generalized additive model assuming a Poisson distribution and using an isotropic spline to describe the spatial variation of the different indicators [7]. The level of smoothness of the spatial terms was selected using the restricted maximum likelihood method.

Finally, we used a Poisson regression model for the univariate and multivariate analyses of risk factors for cholera morbidity and mortality, and present here crude and adjusted relative risks (RR, ARR) and associated 95% confidence intervals. The district of Plaisance was considered as the reference for comparisons among districts.

## Results

### General description

The survey took place from April 22^nd^ to May 13^th^ 2011. In total, 138 randomly selected clusters were visited, and information on 3,187 households and 16,946 individuals collected, which corresponded to approximately 9% of the area’s estimated rural population (173,903 inhabitants). Of these, 46 individuals were subsequently excluded from the analysis due to incorrect inclusion criteria (n = 28) or missing data (n = 18). The median number of individuals per household was 5 (range 1 to 20 persons). The male/female ratio was 0.91 and the median age was 21 years (IQR: 11–40).

**Fig 2:**
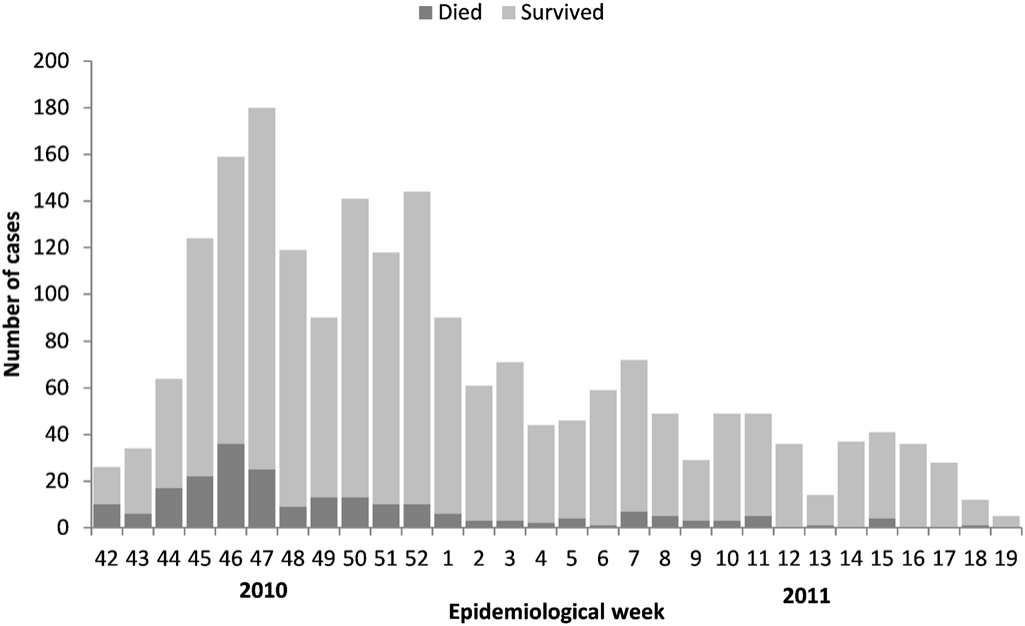
Number of diarrhea cases and deaths reported in the survey per week during the recall period, Nord Department, Haiti, November 2010-March 2011.

In total, 2,034 persons (12.0%) reported at least one episode of watery diarrhea during the recall period (Fig. 2). Among them, 1,979 (97.3%, 95% CI: 96.0–98.2) reported a single episode during the recall period (range 1–4). The median length of the most severe episode investigated in each individual who reported watery diarrhea was 3 days (IQR: 2–4; range 1–15). Of those individuals reporting diarrhea, 68.9% (95% CI: 63.1–74.1) reported vomiting, and 38.3% (95% CI: 33.5–43.4) lethargy or altered consciousness during the diarrheal illness, and were therefore considered as severe cases.

### Attack rate

The attack rate of watery diarrhea in the area during the recall period was 12.0% (95% CI: 10.8–13.2), with a design effect of 5.9. Extrapolated to the rural population in the four districts, this translated into an estimate of 21,681 individuals (95% CI: 19,440–23,922) suffering from watery diarrhea during the recall period.

**Fig 3:**
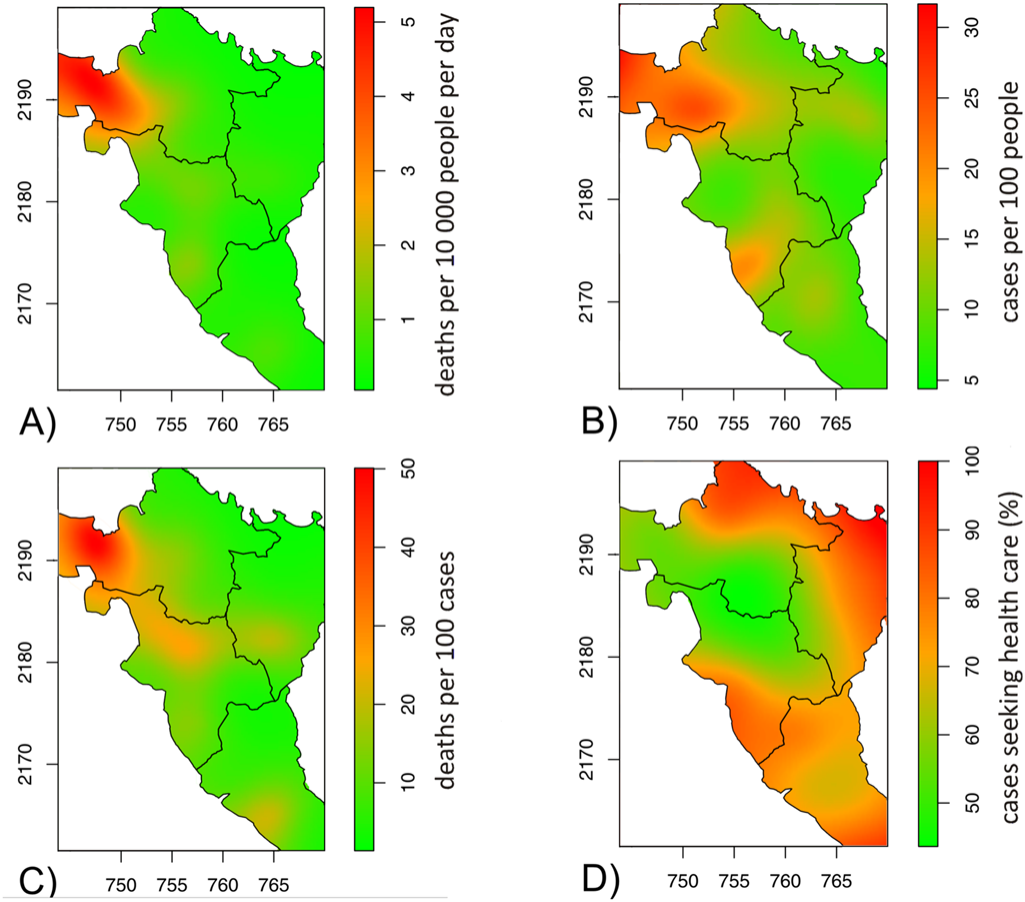
Geographical distribution of crude mortality rate (A), acute watery diarrhea attack rate (B), acute watery diarrhea case-fatality rate (C), health-seeking behavior of acute watery diarrhea case-patients (D), in the Nord Department, Haiti, November 2010-March 2011. The models were fitted in a general additive model framework using Poisson regression with smoothing splines. The spatial term was significant for the four indicators with p-values<0.001. The values on the x and y axis represent the UTM coordinates.

The geographical distribution of attack rates showed marked disparities, with attack rates estimated at more than 20% in some sections in the west of Borgne and Pilate and lower than 10% in most sections of the Plaisance and Port Margot districts (Fig. 3). This was reflected in the estimated attack rates by district, which ranged from 8.6% in Port Margot to 16.2% in Borgne (Table 1).

**Table 1:** Main indicators by district and adjusted risk ratios in Nord Department, Haiti, November 2010-March 2011.

	%	95% CI	ARR*	95% CI	p-value
Attack rate					
Plaisance	9.6	[7.8–11.8]	Ref		
Port Margot	8.6	[7.1–10.3]	0.89	[0.67–1.17]	0.383
Pilate	12.0	[9.7–14.6]	1.24	[0.93–1.66]	0.152
Borgne	16.2	[14.2–18.5]	1.72	[1.34–2.19]	**<0.001**
Case fatality rate					
Plaisance	5.5	[2.9–10.0]	Ref		
Port Margot	5.3	[2.4–11.0]	0.94	[0.35–2.50]	**0.897**
Pilate	11.8	[8.7–15.8]	2.14	[1.06–4.33]	0.035
Borgne	15.2	[10.6–21.4]	2.70	[1.33–5.5]	0.007
Crude mortality rate (per 10,000 per day)					
Plaisance	0.42	[0.2–0.7]	Ref		
Port Margot	0.33	[0.2–0.6]	0.76	[0.32–1.82]	0.533
Pilate	0.90	[0.6–1.3]	2.07	[1.05–4.08]	**0.035**
Borgne	1.46	[1.0–2.1]	3.57	[1.79–7.12]	**<0.001**
Health-seeking					
Plaisance	74.6	[66.8–81.1]	Ref		
Port Margot	83.2	[74.8–89.2]	1.12	[0.98–1.28]	**0.073**
Pilate	77.7	[70.0–83.8]	1.04	[0.92–1.18]	**0.536**
Borgne	61.6	[53.3–69.3]	0.83	[0.71–0.98]	0.024

* ARR, adjusted risk ratio. Adjusted by age and sex using Plaisance as the reference district

### Crude mortality and case fatality rates

In total, 275 individuals were reported to have died during the recall period, leading to a crude mortality rate estimate of 0.82 deaths per 10,000 persons per day (95% CI: 0.64–1.05), which represented 1.62% (95% CI: 1.26–2.07) of the population during the recall period or 2,925 (95% CI: 2199–3651) deaths of all causes when extrapolated to the rural population of the four districts (Plaisance: 393; Port Margot: 246; Pilate: 746; Borgne: 1540). Most of these deaths (84.8%; 95% CI: 77.5–90.0) were attributed to diarrhea.

Of the 2,034 diarrhea cases, the outcome of the episode was death in 224, for a CFR of 11.0% (95% CI: 8.6–13.9) with a design effect of 3.8. Extrapolated to the rural population of the four districts, this represents 2,375 (95% CI: 1,710–3,040) deaths due to diarrhea during the recall period (Plaisance 256; Port Margot 155; Pilate 609; Borgne 1,355).

The overall CFR in both Borgne and Pilate was significantly higher than in Plaisance, the reference district (Table 1). The highest CFR (up to 30–40%) were found in western Borgne and Pilate (Fig. 3).

### Health-seeking behavior

Of 2,030 individuals reporting diarrhea and for whom information on health-seeking behavior was available, 1,447 (71.2%, 95% CI: 66.3–75.6) sought care in a health structure. More than 50% of those who sought care visited a specialized CTC or CTU, and only 3% reported using the ORPs (Table 2). Overall, the main reasons for not seeking care were that the health structure was too far or that the diarrhea was not perceived as requiring care or not perceived as cholera. Among the most severe cases, almost two-thirds reported distance as the main reason for not seeking care (Table 2).

**Table 2:** Health-seeking behavior among all diarrhea cases versus severe cases, Nord Department, Haiti, November 2010-March 2011.

	All (N = 2030)			Severe cases (N = 776)		
	n	%	95% CI	n	%	95% CI
**Sought care**	**1447**			**599**		
MSF CTC or CTU	773	53.3	[47.0–59.6]	358	59.6	[51.0–67.6]
Hospital	508	35.0	[29.5–40.9]	170	28.3	[22.2–35.4]
Non-MSF CTC or CTU	101	7.2	[4.8–10.7]	60	10.3	[6.4–16.0]
ORP	44	3.0	[1.2–7.4]	24	4.0	[1.2–12.4]
Doctor	16	1.1	[0.4–2.8]	2	0.3	[0.0–2.4]
Traditional medicine	19	1.3	[0.6–2.6]	2	0.3	[0.0–1.4]
Other	6	0.4	[0–2.9]	0	0	
**Did not seek care**	**587**			**177**		
Distance	216	36.5	[28.2–45.8]	109	61.5	[16.8–74.4]
No perceived need	172	29.6	[22.8–37.4]	16	9.2	[4.8–16.9]
Illness not perceived as cholera	100	17.0	[11.6–24.4]	25	14	[7.8–24.0]
Too expensive	48	8.2	[5.2–12.7]	9	5.2	[2.4–10.7]
Ashamed	22	3.8	[2.0–7.1]	8	4.6	[1.8–11.5]
Did not know where to go	13	2.2	[1.1–4.3]	5	2.8	[0.8–9.6]
Busy	3	0.5	[0.2–1.6]	1	0.6	[0.0–3.9]

*Respondents were allowed to give more than one reason

The lowest proportion of individuals seeking care was in the remote areas of western Borgne and Pilate (Fig. 3). Of the four districts, the highest proportion of patients seeking care was in Port Margot (83.2%) and lowest in Borgne (61.6%) (Table 1). The reasons for not seeking care also varied by district: distance was cited as a barrier by 52.7% of patients who did not seek care in Borgne but was less cited in Pilate (20.8%), Plaisance (14.5%) and Port Margot (13.5%). In the latter two districts, the main reason for not seeking care was a combination of no perceived need and illness not perceived as cholera (Plaisance: 59.7%; Port Margot: 55.7%, Pilate: 41.4%, Borgne: 42.7%).

### Risk factors for diarrhea-associated mortality

A stratified analysis of risk factors by district showed that similar factors contributed to higher CFR across all districts: older age (> = 60 years), greater severity of illness, living in remote areas, and not seeking health care (Table 3). These factors were also found to have a significant association in the univariate analysis (Table 4). Factors associated with highest risk were severity of disease (RR = 8.1) and not seeking care (RR = 5.1). There was no significant difference in case fatality between males and females.

**Table 3:** Case fatality rate by district and stratified by age groups, sex, severity, mode of transport and health seeking behavior, Nord Department, Haiti, November 2010-March 2011.

	Plaisance		Port Margot		Pilate		Borgne	
	% CFR	95% CI	% CFR	95% CI	% CFR	95% CI	% CFR	95% CI
Age groups								
<5 years	1.5	0.2–10.5	6.7	0.8–39.3	3.8	0.9–15.1	10.4	4.4–22.6
6–9 years	6.4	0.9–34.8	0.0		13.1	4.7–31.4	9.1	3.7–20.6
10–19 years	1.2	0.2–8.5	1.9	0.3–12.0	9.2	4.0–22.9	7.6	3.9–14.3
20–29 years	1.0	0.1–7.5	5.0	1.2–19.3	10.1	4.0–22.9	10.9	4.6–23.7
30–39 years	8.9	2.9–24.3	0.0		13.3	6.4–25.6	18.5	11.1–28.2
40–49 years	11.4	3.1–33.7	6.6	1.6–23.6	7.5	2.6–19.8	13.6	6.7–25.8
50–59 years	2.6	0.4–15.2	5.0	0.6–31.8	15.9	7.6–30.4	11.5	5.7–21.9
> = 60 years	13.9	7.6–24.1	11.3	3.7–29.4	20.2	12.7–30.5	35.1	22.9–49.7
Sex								
Male	6.4	3.3–11.9	1.5	0.3–6.6	14.1	9.6–20.3	14.7	9.9–21.1
Female	4.6	1.9–10.5	9.0	4.3–17.8	9.7	6.1–15.2	15.7	10.5–22.8
Severity								
Not severe	1.0	0.4–3.0	0.0		4.3	2.0–9.2	4.0	2.2–7.3
Severe	15.7	8.0–28.3	11.0	4.9–22.9	26.4	19.0–35.5	30.9	20.6–43.5
Mode of transport								
Mostly motorbike/car	5.3	2.6–10.4	4.8	1.9–11.5	10.6	7.2–15.3	3.5	1.4–8.5
By foot	9.1	7.9–10.3	13.3	13.3–13.3	16.7	9.5–27.7	18.3	12.7–25.6
Health seeking behavior								
Visited a health structure	3.4	2.7–10.0	1.8	0.7–4.7	7.9	4.7–13.0	5.2	2.9–9.1
Did not visit	11.5	4.0–29.2	20.2	7.2–45.2	25.5	15.3–39.4	31.4	19.0–47.3

**Table 4:** Risk factor analysis for diarrhea-associated case-fatality rate Nord Department, Haiti, November 2010-March 2011.

	RR	p-value	ARR*	p-value	ARR†	p-value	ARR‡	p-value
District								
Plaisance	Ref		Ref		Ref			
Port Margot	0.96	0.93	0.68	0.40	0.81	0.57	0.70	0.44
Pilate	2.15	**0.028**	1.95	**0.038**	1.93	**0.021**	1.64	0.14
Borgne	2.77	**0.006**	2.18	**0.022**	1.49	0.15	1.18	0.58
Age groups								
< 5 years	0.74	0.37	0.77	0.38	0.82	0.41	0.78	0.40
5–59 years	Ref		Ref		Ref		Ref	
> 60 years	2.69	**<0.001**	2.25	**<0.001**	1.57	**0.001**	2.22	**<0.001**
Sex								
Male	Ref		Ref		Ref		Ref	
Female	1.03	0.77	1.10	0.37	1.16	0.10	1.07	0.53
Severity								
Not severe	Ref		Ref		Ref		Ref	
Severe	8.21	**<0.001**	7.59	**<0.001**	9.48	**<0.001**	7.42	**<0.001**
Health seeking behavior								
Visited a health structure	Ref				Ref			
Did not visit	5.12	**<0.001**			5.71	**<0.001**		
Mode of transport								
Mostly motorbike/car	Ref						Ref	
By foot	2.73	**<0.001**					2.20	**0.001**

*Multivariate analysis including commune, age groups, sex, and severity

† Multivariate analysis including commune, age groups, sex, severity and health-seeking behavior

‡ Multivariate analysis including commune, age groups, sex, severity and mode of transport

Stepwise introduction of risk factors in a multivariate analysis showed that the differences between districts remained significant when adjusted for age and severity of disease. Due to colinearity between the two variables, remoteness and health-seeking behavior were introduced separately in the model. In each model, older age, severity of disease, and health-seeking behavior or remoteness were associated independently with a higher risk of death (Table 4). Interestingly, when remoteness was introduced in the model, the differences between districts were no longer significant.

## Discussion

The results of this large community-based survey on the burden of cholera during the first six months of the outbreak in a rural and mountainous area in the northern part of Haiti show very high attack rates and case fatality rates. It highlights important geographical disparities in the four districts investigated, and in particular, the higher risk of both disease and death in the most remote areas. Both the attack rate and case fatality rate found through the survey were more than four times higher than those calculated using data recorded by the national surveillance system in the same period in the Nord department. Moreover, the extrapolated number of cases in the rural populations of these four communes only (21,681 for a population of 173,903) almost reached the total number of cases reported in the national surveillance for the whole department until May 22^nd^ (29,295 for a population of 1,004,247), while the extrapolated number of diarrhea-related deaths in the four communes (2,375) was 3.5 times higher than the total number of deaths (654) reported in the whole department over the same period. This acute underreporting of cases and deaths through the national surveillance system derived from health facility-based cases highlights the importance of community data to better estimate disease burden in areas where national surveillance system may encounter major limitations due to the limited access of the population to health structures. Such data are crucial for targeting the most urgent responses to the highest-priority areas. To achieve this goal, local social leaders (head of villages, religious leaders, etc.) and associations should be mobilized early on to participate in both sensitization and community-based surveillance.

Very remote areas were particularly affected by the outbreak, in terms of both number of cases (high attack rates) and diarrhea-related deaths (high CFR). This led to extremely high mortality rates estimates which suggest that up to 5% of the populations in these areas may have died during the first months of the epidemic. Rural areas are generally thought to show lower attack rates than urban and more crowded areas. For example, MSF generally projects attack rates of 0.2%-1 in rural and 1–5% in urban settings, based on a review of MSF programs in previous cholera epidemics [8]. Our data, as well as others suggest that these estimates should be revised [9,10].

In all districts, CFR were particularly high in elderly people (> 60 years old), in patients with severe diarrhea, those living in remote areas accessible only by foot, and those who did not seek care. In a multivariate analysis, older age, severe diarrhea and not seeking care were independently associated with an increased risk of death. We did not find any association with sex, as reported in other studies, while other risk factor identified elsewhere, such as larger household sizes and being in poor health at onset of disease were not investigated here [11,12]. Not seeking care, in contrast, was reported in all studies with a similar adjusted odds ratio of 5.4 in Guinea Bissau. Health-seeking behavior was influenced by the type of information received in Zimbabwe, with person-to person communication by village health workers being more efficient than other sources of information such as friends, family, NGOs or radio [12]. Here, distance rather than lack of information seemed to be the main barrier to health-care seeking in remote areas.

Accordingly, in a separate multivariate model, remoteness was also independently associated with an increased risk of death, with an adjusted risk ratio of 2.20. In addition, in this model, the differences between districts became non-significant, while the risk of death remained higher in Pilate than Plaisance in the multivariate analysis including health-seeking behavior. This finding suggests that in Borgne and Pilate the mortality risk for diarrhea patients (which was more than twice that in Plaisance), could be explained mostly by the fact that these districts have more remote areas accessible only by foot. In addition to reducing the proportion of people seeking care, the delay to reach the treatment center probably also influenced the risk of dying as suggested by the higher, though not significantly, risk of dying in patients consulting more than 12 hours after onset of diarrhea in Guinea Bissau [11].

Considering their high vulnerability, it is important to improve response strategies for remote populations. Rapid implementation of ORPs in remote settings might be a good option. Here, only a small proportion (3%) of diarrhea cases reported using them. We did not investigate reasons for this low attendance, but their late implementation certainly did reduce their efficacy. Other factors such as low awareness of their purpose and location or lack of confidence in the quality of care provided could have participated. Early community involvement could probably improve all these aspects [13]. Mass vaccination campaigns have shown good efficacy to prevent cholera cases [14] and these would be particularly relevant in remote areas where other prevention or treatment strategies are difficult to sustain. Finally, these data illustrate the lack of adequate general health system in rural areas of Haiti, as well as in many other low and middle-income countries. Improving general access to care in these areas would probably be the best step towards reducing the high burden of cholera outbreaks as well as other diseases.

The main limitation of this retrospective survey may have been recall bias, particularly due to the long recall period. In contrast to mortality data, reporting of diarrhea is highly prone to recall bias in infants, and long recall periods are generally not recommended to assess diarrhea [15,16]. However, diarrhea in adults, particularly severe diarrhea, is rare and thus less prone to recall bias and we believe that it was not a major bias in the reported diarrhea cases. This belief is reinforced by the shape of the epidemic curve obtained through the survey, which is similar to those reported by the national surveillance system (http://mspp.gouv.ht). However, information bias might have had a more important impact on respondents’ report of the health structures visited and could have led in particular to an under-estimation of the number of visits to an ORP if patients also visited a higher-level health structure. Another limitation of our risk factor analysis was that it was a post-hoc analysis and we did not explore all risk factors, such as access to water and sanitation, socio-economic status, or access to health information.

In conclusion, we show here that attack rates and case fatality rates of the first cholera epidemic peak were much higher than reported by the national surveillance system, and that people living in very remote areas in the Nord department were particularly at risk for both disease and death during the early phase of the outbreak. Although an initial response focusing on urban and more densely populated areas was appropriate considering the large number of patients treated, this analysis shows that rural areas with poor access to health care and to cholera prevention and treatment information were at the greatest risk. Adapted strategies to rapidly provide access to preventive activities and treatment in remote communities are urgently needed to prevent this disproportionate impact in future cholera outbreaks.

## Supporting Information

S1 Checklist STROBE checklist. (DOCX)

S1 Dataset Study dataset. (XLSX)
