# Supplementary material for: Geographic Distribution and Mortality Risk Factors during the Cholera Outbreak in a Rural Region of Haiti, 2010-2011
Source: PLoS Negl Trop Dis. 2015 Mar 26;9(3):e0003605. doi: 10.1371/journal.pntd.0003605 (PMC4374668; doi:10.1371/journal.pntd.0003605)
Supplement: S1 Checklist — (DOCX) [file pntd.0003605.s001.docx]

STROBE Statement—checklist of items that should be included in reports of observational studies

|  | Item No. | Recommendation | Page  No. | Relevant text from manuscript |
| --- | --- | --- | --- | --- |
| **Title and abstract** | 1 | (*a*) Indicate the study’s design with a commonly used term in the title or the abstract | 2 | “A two-stage, household based cluster survey was conducted…” |
|  |  | (*b*) Provide in the abstract an informative and balanced summary of what was done and what was found | 2 | See abstract |
| Introduction | | | |  |
| Background/rationale | 2 | Explain the scientific background and rationale for the investigation being reported | 4 | “Since national surveillance data were based on reports from the health structures and were likely to miss community cases, large retrospective population-based surveys were conducted by Médecins Sans Frontières (MSF)” |
| Objectives | 3 | State specific objectives, including any prespecified hypotheses | 4 | “…to estimate the cholera burden during the first weeks of the epidemic and get insight into health-seeking behavior.”  “..a survey that was conducted in a large rural, mountainous area across four districts of the Nord department, chosen to facilitate comparison between regions with good versus poor accessibility by road, and with rapid versus delayed response to the outbreak.” |
| Methods | | | |  |
| Study design | 4 | Present key elements of study design early in the paper | 6 | See Study design section |
| Setting | 5 | Describe the setting, locations, and relevant dates, including periods of recruitment, exposure, follow-up, and data collection | 5,6,8 | See Study setting section for study setting and location  Dates: “a recall period of 170 days (from October 17^th^, 2010 to the earliest survey date)” (p.6)  “The survey took place from April 22^nd^ to May 13^th^ 2011.” (P.8) |
| Participants | 6 | (*a*) *Cohort study*—Give the eligibility criteria, and the sources and methods of selection of participants. Describe methods of follow-up  *Case-control study*—Give the eligibility criteria, and the sources and methods of case ascertainment and control selection. Give the rationale for the choice of cases and controls  *Cross-sectional study*—Give the eligibility criteria, and the sources and methods of selection of participants | 6 | Selection of households: “The corresponding GPS points were used in the field to locate the initial house of each cluster. The next house was selected by proximity, i.e., next closest house, until 23 households had been visited in each cluster.”  Definition of participants: :”the head of household was asked to provide the age and sex of all household members (defined as persons living under the same roof and sharing meals)” |
|  |  | (*b*) *Cohort study*—For matched studies, give matching criteria and number of exposed and unexposed  *Case-control study*—For matched studies, give matching criteria and the number of controls per case |  | NA |
| Variables | 7 | Clearly define all outcomes, exposures, predictors, potential confounders, and effect modifiers. Give diagnostic criteria, if applicable | 6-7 | “ … episodes of diarrheal illness (defined as at least three watery stools within a 24-hour period) and deaths that occurred during the recall period”  “Information collected included duration and symptoms of the episode, health-seeking behavior (i.e., type(s) of health structure(s) visited or reason for not visiting a health structure), and outcome (i.e., death or survival). Severe cases were defined as those in which patients reported lethargy or altered consciousness during the diarrheal illness. Death was considered related to diarrhea when it was reported as the outcome of the most severe diarrheal episode.  In each cluster, the time and type of transport to the closest village with a health structure (excluding ORPs) was documented. “ |
| Data sources/ measurement | 8* | For each variable of interest, give sources of data and details of methods of assessment (measurement). Describe comparability of assessment methods if there is more than one group | 6 | “For each household member present at the beginning of the recall period, the head of household was asked about….” |
| Bias | 9 | Describe any efforts to address potential sources of bias | 7 | “As not all clusters achieved a sample of 23 households, weighted analysis was used to adjust for the probability of each household being selected, by dividing the expected household number per cluster (23) by the actual number of households included. In all analyses, we accounted for the clustering of households within the cluster and applied the selection weights. Design effects are reported where relevant.” |
| Study size | 10 | Explain how the study size was arrived at | 6 | “The sample size was 16,000 individuals, calculated to estimate an expected crude mortality rate of 0.5 per 10 000 persons per day with a precision of 0.1, an anticipated design effect of two and a recall period of 170 days” |

Continued on next page

| Quantitative variables | 11 | Explain how quantitative variables were handled in the analyses. If applicable, describe which groupings were chosen and why |  | NA |
| --- | --- | --- | --- | --- |
| Statistical methods | 12 | (*a*) Describe all statistical methods, including those used to control for confounding | 7 | See Statistical analysis |
|  |  | (*b*) Describe any methods used to examine subgroups and interactions | 7 | “Finally, we used a Poisson regression model for the univariate and multivariate analyses of risk factors for cholera morbidity and mortality, and present here crude and adjusted relative risks (RR, ARR) and associated 95% confidence intervals. The district of Plaisance was considered as the reference for comparisons among districts.” |
|  |  | (*c*) Explain how missing data were addressed | 8 (Results) | “Of these, 46 individuals were subsequently excluded from the analysis due to incorrect inclusion criteria (n=28) or missing data (n=18)”. |
|  |  | (*d*) *Cohort study*—If applicable, explain how loss to follow-up was addressed  *Case-control study*—If applicable, explain how matching of cases and controls was addressed  *Cross-sectional study*—If applicable, describe analytical methods taking account of sampling strategy | 7 | “In all analyses, we accounted for the clustering of households within the cluster and applied the selection weights.” |
|  |  | (*e*) Describe any sensitivity analyses | NA |  |
| Results | | | | |
| Participants | 13* | (a) Report numbers of individuals at each stage of study—eg numbers potentially eligible, examined for eligibility, confirmed eligible, included in the study, completing follow-up, and analysed | 8 | “..information on 3,187 households and 16,946 individuals collected..”  “..46 individuals were subsequently excluded from the analysis..” |
|  |  | (b) Give reasons for non-participation at each stage | 8 | See above for those excluded from the analysis. |
|  |  | (c) Consider use of a flow diagram |  | Considering the study design, there was little use of including a flow diagram |
| Descriptive data | 14* | (a) Give characteristics of study participants (eg demographic, clinical, social) and information on exposures and potential confounders | 8 | “The male/female ratio was 0.91 and the median age was 21 years (IQR: 11-40).” |
|  |  | (b) Indicate number of participants with missing data for each variable of interest |  | Participants with missing data on key variable were excluded from the analysis |
|  |  | (c) *Cohort study*—Summarise follow-up time (eg, average and total amount) |  | NA |
| Outcome data | 15* | *Cohort study*—Report numbers of outcome events or summary measures over time |  | *NA* |
|  |  | *Case-control study—*Report numbers in each exposure category, or summary measures of exposure |  | *NA* |
|  |  | *Cross-sectional study—*Report numbers of outcome events or summary measures | 8,9 | “In total, 2,034 persons (12.0%) reported at least one episode of watery diarrhea during the recall period”  “In total, 275 individuals were reported to have died during the recall period”  “Of the 2,034 diarrhea cases, the outcome of the episode was death in 215,” |
| Main results | 16 | (*a*) Give unadjusted estimates and, if applicable, confounder-adjusted estimates and their precision (eg, 95% confidence interval). Make clear which confounders were adjusted for and why they were included | 8,9  Table 1  Table 4 | See global estimates of attack rate, crude mortality rate, case fatality rate, and health-seeking behavior and their 95% CI in the text  See estimates (and their 95% CI) by commune in Table 1.  See crude and adjusted risk ratios for CFR in Table 4 |
|  |  | (*b*) Report category boundaries when continuous variables were categorized |  | NA |
|  |  | (*c*) If relevant, consider translating estimates of relative risk into absolute risk for a meaningful time period | 8,9 | The estimates of attack rate and crude mortality rate were used to extrapolate the number of cases and diarrhea-related deaths to the overall target population during the recall period:  “Extrapolated to the rural population in the four districts, this translated into an estimate of 21,681 individuals (95% CI: 19,440 – 23,922) suffering from watery diarrhea during the recall period.”  “..or 2,925 (95% CI: 2199-3651) deaths of all causes when extrapolated to the rural population of the four districts” |

Continued on next page

| Other analyses | 17 | Report other analyses done—eg analyses of subgroups and interactions, and sensitivity analyses | Figure 3 | See geographical analysis in Figure 3 |
| --- | --- | --- | --- | --- |
| Discussion | | | | |
| Key results | 18 | Summarise key results with reference to study objectives | 10 | “The results of this large community-based survey on the burden of cholera during the first six months of the outbreak in a rural and mountainous area in the northern part of Haiti show very high attack rates and case fatality rates. It highlights important geographical disparities in the four districts investigated, and in particular, the higher risk of both disease and death in the most remote areas.” |
| Limitations | 19 | Discuss limitations of the study, taking into account sources of potential bias or imprecision. Discuss both direction and magnitude of any potential bias | 12 | See paragraph on limitations starting with “The main limitation, ….” |
| Interpretation | 20 | Give a cautious overall interpretation of results considering objectives, limitations, multiplicity of analyses, results from similar studies, and other relevant evidence | 12 | “In conclusion, we show here that attack rates and case fatality rates of the first cholera epidemic peak were much higher than reported by the national surveillance system, and that people living in very remote areas in the Nord department were particularly at risk for both disease and death during the early phase of the outbreak.” |
| Generalisability | 21 | Discuss the generalisability (external validity) of the study results | 12 | The main objective of this article is to report on the first months of the cholera outbreak in Haiti, which cannot be generalized. However, an effort was made to discuss how these results could be used to avoid such a high burden in remote populations elsewhere in future outbreaks. See the paragraph on p.12 starting with “Considering their high vulnerability, it is important to improve response strategies for remote populations…” |
| Other information | |  | | |
| Funding | 22 | Give the source of funding and the role of the funders for the present study and, if applicable, for the original study on which the present article is based |  | Sources of funding were provided on the PloS NTD website as requested |

*Give information separately for cases and controls in case-control studies and, if applicable, for exposed and unexposed groups in cohort and cross-sectional studies.

**Note:** An Explanation and Elaboration article discusses each checklist item and gives methodological background and published examples of transparent reporting. The STROBE checklist is best used in conjunction with this article (freely available on the Web sites of PLoS Medicine at http://www.plosmedicine.org/, Annals of Internal Medicine at http://www.annals.org/, and Epidemiology at http://www.epidem.com/). Information on the STROBE Initiative is available at www.strobe-statement.org.
